# Viral hepatitis amongst Saharia: a primitive tribe of Madhya Pradesh

**DOI:** 10.1186/1471-2334-12-S1-P61

**Published:** 2012-05-04

**Authors:** VG Rao, J Bhat, R Yadav

**Affiliations:** 1 Regional Medical Research Centre for Tribals (RMRCT), (Indian Council of Medical Research), Jabalpur - 482 003, India

## Background

The prevalence of viral hepatitis in tribal areas of India mostly remains unknown. Saharia is one of the primitive tribes of Madhya Pradesh and live primarily in the Sheopur and Shivpuri districts. The purpose of the present study was to assess the prevalence of hepatitis viruses in Saharia tribal community of central Indian state of Madhya Pradesh.

## Methods

A cross sectional study was carried out to determine the point prevalence of different hepatitis viruses amongst Saharia primitive tribal community of Madhya Pradesh, central India. After obtaining the informed consent, blood samples (5 ml each) were collected from them. Serum was separated on site, aliquoted and transported to the laboratory maintaining the cold chain. The markers of various hepatitis viruses were detected using commercial ELISA kits. The specimens found positive for HBsAg were tested for HBV DNA by Polymerase Chain Reaction (PCR).

## Results

A total of 173 blood samples were collected. The prevalence of HBsAg and anti HBs was found to be 5% and 33% respectively. The prevalence of anti HCV was 1%. Anti HAV antibodies were present in 165 samples (99%). The prevalence of anti HEV was found to be 40%. 4 samples were found positive for HBV DNA by real time PCR.

## Conclusion

The findings of the study indicate that viral hepatitis infection is an important problem in saharia primitive tribal community. Control measures including IEC strategies are necessary among them.

